# Genomic Profiling Reveals That Transient Adipogenic Activation Is a Hallmark of Mouse Models of Skeletal Muscle Regeneration

**DOI:** 10.1371/journal.pone.0071084

**Published:** 2013-08-15

**Authors:** Laura Lukjanenko, Sophie Brachat, Eliane Pierrel, Estelle Lach-Trifilieff, Jerome N. Feige

**Affiliations:** MusculoSkeletal Diseases Group, Novartis Institutes for Biomedical Research, Basel, Switzerland; IRCCS-Policlinico San Donato, Italy

## Abstract

The marbling of skeletal muscle by ectopic adipose tissue is a hallmark of many muscle diseases, including sarcopenia and muscular dystrophies, and generally associates with impaired muscle regeneration. Although the etiology and the molecular mechanisms of ectopic adipogenesis are poorly understood, fatty regeneration can be modeled in mice using glycerol-induced muscle damage. Using comprehensive molecular and histological profiling, we compared glycerol-induced fatty regeneration to the classical cardiotoxin (CTX)-induced regeneration model previously believed to lack an adipogenic response in muscle. Surprisingly, ectopic adipogenesis was detected in both models, but was stronger and more persistent in response to glycerol. Importantly, extensive differential transcriptomic profiling demonstrated that glycerol induces a stronger inflammatory response and promotes adipogenic regulatory networks while reducing fatty acid β-oxidation. Altogether, these results provide a comprehensive mapping of gene expression changes during the time course of two muscle regeneration models, and strongly suggest that adipogenic commitment is a hallmark of muscle regeneration, which can lead to ectopic adipocyte accumulation in response to specific physio-pathological challenges.

## Introduction

Skeletal muscle is a highly plastic tissue, which responds to exercise or disuse by modulating the mass and composition of contractile proteins [1], [2]. In addition, muscle fibers have a strong regenerative capacity as muscle injuries trigger the proliferation and activation of satellite cells, a specific type of stem cells expressing the marker Paired box protein 7 (Pax7) and committed to the myogenic lineage [3]–[5], which subsequently fuse to injured fibers to promote their efficient repair. Other cell types are also involved in muscle repair during the different phases of the healing process [4], [6]. In particular, immune cells are recruited to degenerating muscle to allow the removal of cellular debris and support myogenesis [7], [8]. In addition, a novel type of Pax7-negative myogenic progenitors expressing the marker PW1 also participate to muscle regeneration [9]. Despite the ability of healthy skeletal muscle to regenerate, several pathological conditions such as muscular dystrophies or aging impair satellite cell homeostasis and myofiber regeneration [10], [11], thereby weakening muscle plasticity and integrity. In such diseases, excessive cycles of degeneration/regeneration prime the muscle for fibrosis and ectopic adipocyte accumulation, leading to an exhaustion of the regenerative capacity and ultimately to impaired muscle contraction.

Muscle ectopic adipogenesis is particularly prominent in myopathies such as Duchenne muscular dystrophy, where young boys with dystrophin mutations have important fat infiltration that can reach up to 50% of muscle content in the gluteus muscle [12]. Intra-muscular fat accumulation also occurs in sarcopenia where marbling of skeletal muscle by adipose tissue plays an important role in contractile and metabolic dysfunction [13], [14]. It has been recently demonstrated that fat cells which invade skeletal muscle originate from mesenchymal progenitors distinct from satellite cells and expressing the platelet-derived growth factor receptor alpha (PDGFRα) [15]–[17]. Using lineage tracing, PDGFRα has also recently been recognized as a general marker for adipogenic progenitors giving rise to mature fat cells in white and brown adipose tissues [18], [19]. Interestingly, muscle-resident PDGFRα-positive progenitors can also give rise to collagen-type I expressing cells, indicating that ectopic adipogenesis and fibrosis are regulated in parallel from common fibro/adipogenic progenitors (FAPs) [20], [21]. In order to differentiate into pathological fat or fibrotic depots, FAPs require external triggers, that remain to be characterized, but rely on the muscle environment rather than the progenitors themselves [15], [17]. Human PDGFRα-positive FAPs have also recently been demonstrated to have osteogenic potential, and could contribute to pathological calcification of skeletal muscle occurring during Myositis Ossificans [22]. However, FAPs also seem to positively influence myogenesis and muscle regeneration as they are activated upon muscle damage and show increased expression of IL-6 [20], a factor that promotes myogenesis [4], [23], [24]. In addition, when co-cultured with myogenic progenitors *in vitro*, FAPs induce myogenic differentiation in a dose-dependent manner, suggesting that they might play an active role in muscle regeneration [20]. Furthermore, it has recently been demonstrated that IL-4/IL-13 signaling is activated in FAPs in response to IL-4 secretion by eosinophils upon injury, thus promoting FAP proliferation to support myogenesis and inhibiting their commitment toward adipogenesis [25].

Many mouse models have been developed to study skeletal muscle regeneration, out of which the intramuscular injection of toxins such as cardiotoxin (CTX) has been one of the most extensively studied [26]. In contrast, the intramuscular injection of glycerol has recently been recognized as a new model of regeneration which promotes ectopic adipogenesis in muscle [16], [17], [20], [27]. Although the model of intramuscular glycerol injection has been technically refined recently [16], understanding the causes and mechanisms of ectopic fat cell deposition remains an open question. Towards that goal, we conducted a comprehensive profiling of the molecular and histological responses occurring after muscle damage induced by CTX and glycerol at 4 different time points. Our results demonstrate that the myogenic response overlaps to a large extent in both injury models. Surprisingly, an adipogenic response was also detected in both models, although glycerol induced stronger and more prolonged adipocyte formation. Altogether, our data provide a comprehensive correlation between the molecular and histological changes differentially occurring during glycerol and CTX regeneration, and the transcriptional signatures of these two injury models constitute a key resource to further understand muscle regeneration and ectopic adipogenesis.

## Materials and Methods

### Animals

All animal experiments were approved by the Kantonales Veterinäramt Basel Stadt, Switzerland. Adult C57BL/6J male mice were purchased from The Jackson Laboratory, maintained at 22°C in a 12-h light–12-h dark cycle with unrestricted access to regular diet and water and injured at 12 to 14 weeks old.

### Muscle injury and muscle preparation

25 µl of 50% v/v glycerol or 25 µl of 10 µM cardiotoxin (CTX) was injected through two injections of 12.5 µl into tibialis anterior (TA) muscle, using a 22 gauge needle (Hamilton). The intramuscular injections were performed under anesthesia using isoflurane inhalation and mice were injected intra-peritoneally with 0.1 mg/kg of Buprenorphine (Temgesic), one hour before injury and the day after, as analgesic. Mice were sacrificed at the indicated time points with CO_2_ (air mixture 85∶15) in an inhalation chamber. TA muscles were cut into two parts, one part being frozen into liquid nitrogen for total RNA extraction and the other part being embedded into OCT, frozen for 2 seconds in liquid nitrogen and then frozen in isopentane cooled with liquid nitrogen for histological analysis.

### Immunohistochemistry and microscopy

Frozen muscle tissues were sectioned (10 µm thickness) using a cryostat HM 560 (Microm-Thermo Fisher Scientific Inc). Muscle sections were subjected to hematoxylin-eosin (H&E) staining and the slides were mounted in Pertex (Histolab). Immunostainings were performed using polyclonal antibodies against perilipin or laminin detected using goat anti-rabbit IgG antibodies conjugated to Alexa 488 or 555 (Molecular Probes), respectively. For perilipin immunostaining, cryosections were allowed to dry during 10 minutes, and fixed in PFA 4% during 10 minutes. After 2 quick washes in PBS, samples were permeabilized during 10 minutes in 0.5% Triton X-100 (Sigma-Aldrich) in PBS (0.5% PBTX), and blocked for 1 hour at room temperature in blocking solution (2% Goat Serum (Sigma-Aldrich) in 0.5% PBTX). Cryosections were stained during 3 hours at room temperature using monoclonal anti-perilipin A/B antibody produced in rabbit (Sigma-Aldrich) diluted at 1/250 in the block solution. After 3 washes of 5 minutes in PBS, slides were incubated during 1 hour at room temperature with the secondary antibody diluted at 1/200 in the blocking solutions. Slides were washed again, and mounted in Prolong Gold Medium with DAPI (Invitrogen). For laminin immunostaining, cryosections were allowed to dry during 10 minutes and were permeabilized during 10 minutes in PBS and blocked for 45 minutes at room temperature in the blocking solution (10% Goat Serum (Sigma-Aldrich), 1% bovine serum albumin (BSA) (Sigma) in 0,5% PBTX). Cryosections were stained during 3 hours at room temperature using monoclonal anti-Laminin antibody produced in rabbit (Sigma-Aldrich) diluted at 1/200 in the blocking solution. After 3 washes of 10 minutes in PBS, slides were incubated during 1 hour at room temperature with secondary antibody diluted at 1/200 in the blocking solutions. Slides were washed again, and mounted in Prolong Gold Medium with DAPI (Invitrogen). Stained tissues were photographed using Olympus VS120 Virtual Microscopy Slide Scanning System and analyzed using the VS-ASW FL software measurement tools.

### Image analysis

The number of myofibers with central nuclei was calculated from laminin/DAPI stainings on all fibers of the section using an automated image processing software developed internally (Astoria). Cells expressing perilipin and their surface were counted manually on the whole cryosection.

### RNA extraction & preparation for profiling

Total RNA was extracted from the frozen tibialis part, using an RNeasy Fibrous Tissue Mini Kit (Qiagen), and eluted in 40 μl RNase free water. RNA concentration and quality was measured by a ND-1000 spectrophotometer (NanoDrop, Thermo Scientific). In case RNA quality was not acceptable (ratio 260/230<1.5), samples were cleaned using a RNeasy Plus Micro Kit (Qiagen), eluted in 15 μl and the new concentration and ratios measured again. RNA final quality was assessed on an Agilent 2100 Bioanalyzer using the Agilent RNA 6000 Nano kit, and processed for microarray when RIN>7.

### Microarray processing and data analysis

RNA samples were subjected to microarray analysis on Affymetrix GeneChip Mouse Genome 430 2.0 chips (Affymetrix, Santa Clara, CA) according to the manufacturer's recommendations. All statistical analyses were performed using R/Bioconductor (www.bioconductor.org). Quality control was performed using both AffyQCreport and arrayQCmetrics packages. Data was RMA normalized using RMA and scaled to a 2% trimmed mean of 150. Probes with normalized expression values below 50 in all groups were filtered out. Differential gene expression was performed using a linear model approach (Limma). Genes with a fold change higher than 2 and an adjusted P-value below 0.01 (Benjamini and Hochberg multiple testing correction) were considered regulated. Venn diagrams were drawn using the BioVenn online tool (http://www.cmbi.ru.nl/cdd/biovenn/) [28]. Gene set enrichment analysis (GSEA) [29], was performed on fold change ranked list of all non filtered probesets collapsed to gene symbols [30] using the Broad (www.broadinstitute.org/gsea) and the Molecular Signature v3.0 gene sets databases. GSEA results were further analyzed using the enrichment map tool [31] and visualized in Cytoscape [32]. Other visualizations were performed using Tibco Spotfire. Pathway maps were generated using Pathvisio [33]. Data was submitted to the Gene Expression Omnibus repository and is available under the accession number GSE45577.

### Reverse Transcription and qPCR

Total RNA extracts were diluted at 100 ng/µl and reverse transcribed into cDNA using the High Capacity cDNA Reverse Transcription Kit (Applied Biosystems) and then diluted 1/20. Quantitative PCR reactions were performed on a BioRad thermocycler (association of CFX384^TM^ Real-Time System and C1000^TM^ Thermal Cycler) with HotGoldStar DNA polymerase (qPCR Master Mix Plus, Eurogentec). The amplification curves were analyzed by the BioRad CFX Manager software. Specific Taqman probes (Applied Biosystems by Life Technologies) are listed in Table S1. 18 S was amplified as qPCR normalization control.

### Statistical Analysis of qPCR and histology data

Statistical significance was assessed by the Student's t-test for binary comparisons. For comparison of more than 2 groups, one-way ANOVA followed by Bartlett's test was used. All data are expressed as mean value +/− s.e.m.; and unless otherwise indicated, *n* = 6 in each CTX and glycerol group; and *n = 5* in the control group were analyzed. A p-value smaller than 5% was considered statistically significant.

## Results

### Glycerol and CTX induce similar myofiber damage and degeneration, followed by rapid muscle regeneration

In order to compare the molecular profiles of muscle regeneration after glycerol and CTX injection, the Tibialis Anterior muscle of adult wild-type mice was injected with 25 μl of 50% glycerol or 10 µM CTX and compared to a control muscle 3, 7, 14 or 21 days after injection. The dose of glycerol was selected from a pilot study showing that 25 μl of 50% glycerol was able to induce levels of myofiber damage in a slightly lower, yet comparable range than our established model of CTX-induced degeneration (figure S1) [34].

**Figure 1:**
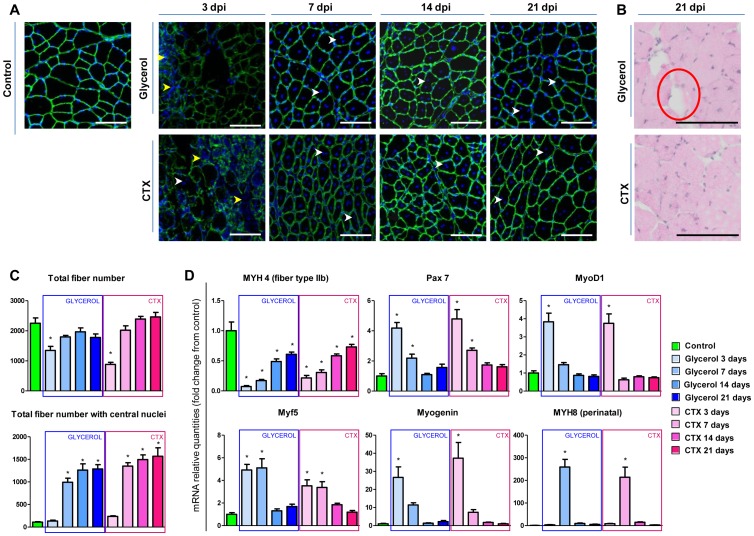
Glycerol and CTX induce similar kinetics of degeneration and regeneration. Control uninjured tibialis anterior muscle, and tibialis anterior muscles injected with either 25 µl of 50% (v/v) glycerol or 10 µM CTX were sectioned and stained with laminin and DAPI 3, 7, 14 or 21 days after injection (dpi) (A), or with hematoxylin-eosin at 21 dpi (B). Cryosections were performed at the mid-belly part of tibialis anterior. Scale bars, 100 μm. Yellow arrow: immune cell nuclei, white arrow: central nuclei, red circle: fat cell-like structure. (C) Quantitative analysis of total myofibers and of myofibers with at least one central nuclei from laminin/DAPI stained sections. (D) qPCR analysis of the mRNA levels of different markers of muscle regeneration. Data are expressed as mean ± s.e.m., n = 5–6/group. * p-value <0.05 *vs*. control. MYH, Myosin Heavy Chain.

As expected, the control muscle was composed of mature differentiated myofibers with multiple nuclei lying exclusively at the periphery of the cell (figure 1A). Both glycerol and CTX injection lead to a complete destruction of myofibers in the degenerating zones at 3 dpi, characterized by a dramatic reduction of the number of myofibers concomitant with an important infiltration of immune cells. Consequently, myofibers started regenerating 7 days post injection in both models, as many newly formed fibers with centrally located nuclei were observed, and the gradual regeneration of myofibers was almost complete after 3 weeks in both models. At 21 dpi, a differential process could be observed between the two models as glycerol-treated mice had much more cellular structures devoid of eosin-positive cytoplasm, which are reminiscent of mature white adipocytes containing triglycerides in a large lipid droplet (figure 1B). In addition, the regeneration capacity seemed slightly better with CTX than with glycerol as the number of regenerating fibers with centralized nuclei increased more rapidly and the total number of fibers recovered more rapidly (figure 1C).

When the molecular profiles of key markers of muscle function and regeneration were measured at the mRNA level, both models initially induced a profound loss of myosin heavy chain IIB (MYH4), which gradually recovered during the regenerative process over 14 to 21 days (figure 1D). As expected, both CTX and glycerol induced Pax7 expression at 3 dpi when satellite cells proliferate in response to muscle injury, and Pax7 levels gradually decreased during regeneration as the new pool of satellite cells engaged in myogenic differentiation and lost the stem cell marker Pax7. Consequently, the downstream myogenic transcription factors MyoD, Myf5 and Myogenin were also strongly induced at 3–7 dpi, but neither Pax7, nor MyoD/Myf5/Myogenin showed differential regulation in CTX vs. glycerol-injected muscles. The embryonic myosin heavy chain MYH8 was also strongly activated at 7 dpi in both models as muscle regeneration transiently re-activates embryonic myogenic programs occurring during development [4], [35]. Altogether, these molecular profiles demonstrate that CTX and glycerol induce similar satellite cell activation and myogenic differentiation.

### *In vivo* ectopic adipogenesis is detected in both models of regeneration but with a stronger amplitude and persistence after glycerol injury

**Figure 2:**
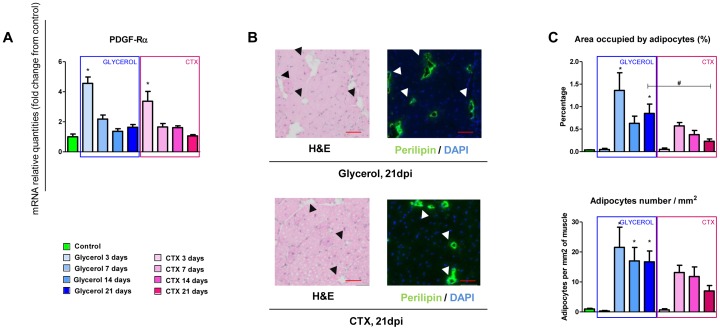
Ectopic adipogenesis occurs in both glycerol- and CTX-induced muscle regeneration. (A) qPCR analysis of the mRNA level of the platelet-derived growth factor receptor alpha (PDGFRα). (B) Cryosections were performed at the mid-belly part of TA and subjected to H&E and perilipin staining at each time points after injection. Representative perilipin (green) /DAPI (blue) fluorescent stainings at 21 dpi are shown next to an H&E staining of the same region. Scale bars, 50 μm. (C), Quantitative analysis of perilipin expression assessed by counting and measuring the area of all perilipin expressing cells per section. Data are expressed as mean ± s.e.m., n = 5–6/group. * p-value <0.05 *vs*. control, # p-value <0.05 in Glycerol *vs*. CTX at same time points.

In order to study ectopic adipogenesis in the two models of muscle regeneration, we first evaluated the expression of the PDGFRα, a marker which is specifically expressed by fibro/adipogenic progenitors (FAPs) [17]. As previously reported [15], [17], [20], PDGFRα mRNA levels increased 3 days after injection of both glycerol and CTX, and returned to the basal expression level at 7 dpi (figure 2A), confirming that FAP activation and proliferation occurs at similar levels after glycerol and CTX injection. Adipogenic differentiation of FAPs was then evaluated through a perilipin staining which labels lipid droplets and allows outlining ectopic adipocytes (figure 2B). As previously reported [16], [17], [36], perilipin expression was detected in the interstitial spaces of muscles treated with glycerol and corresponded to cellular structures devoid of eosin-positive cytoplasm in H&E sections. Surprisingly, ectopic adipocytes also formed during regeneration after CTX injury, suggesting that a transient adipogenic response during muscle regeneration might be more frequent than previously appreciated [17]. Most adipocytes appeared 7 days after glycerol and CTX injections at a similar level, and then slowly decreased in number during muscle regeneration (figure 2C). However, the adipocytes that differentiated in muscles injected with glycerol were generally bigger and persisted for a longer time than in muscles injected with CTX in which the number of fat cells decreased after 7 dpi.

### Glycerol injection regulates more genes than CTX at the genome wide level

**Figure 3:**
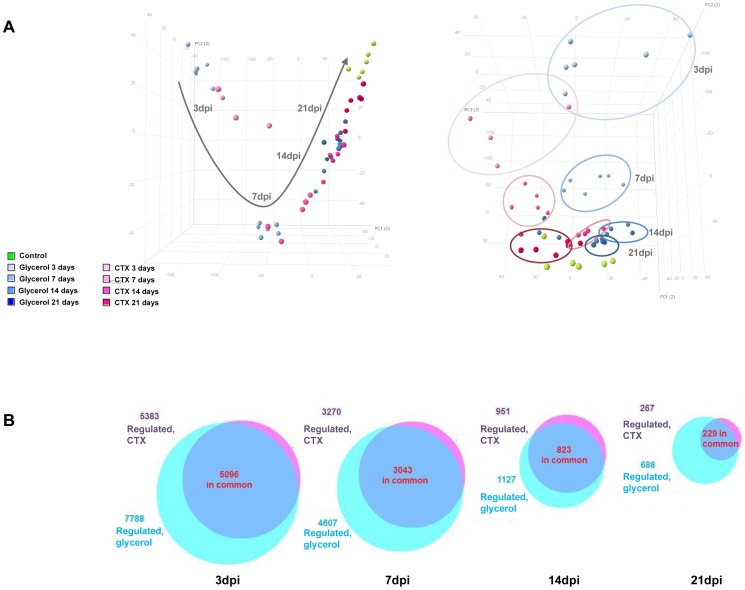
Overview of transcriptomic analysis showing time-dependent genome wide regulation. (A) Principal component analysis diagram showing most variation between time points along the first principal component axis (left graph), and differentiating between models along the third principal component axis (right graph). (B) Venn diagrams comparing the number of regulated genes in the glycerol and CTX models *vs.* control at 3 dpi, 7 dpi, 14 dpi and 21 dpi time points. Gene expression changes were considered significant when the absolute Fold Change >2 and adjusted p-value <0.01.

In order to understand the molecular mechanisms of regeneration and ectopic adipogenesis, we performed a differential profiling of the glycerol and CTX models at the 3, 7, 14 and 21 dpi time points using Affymetrix expression arrays. The numerous variables contributing to inter-sample variations, averaged over all the genes analyzed were reduced through a principal component analysis (PCA) to three variables that account for the major sources of variation in the dataset. Time after injury was the predominant variable, as samples were grouped by time point in the first dimension of the PCA diagram without any distinction between the glycerol and CTX model (figure 3A). The most distant samples in the PCA diagram were the 3 dpi samples, indicating that both glycerol and CTX injections induce a major gene expression change at 3 dpi which likely reflects the massive physiological events and differences in cell-type composition occurring during the first days after injury. Consistently, the number of regulated genes was maximal at 3 dpi (5000–8000 genes, figure 3B). During the regeneration process, samples moved in the PCA space towards the control group while the number of regulated genes gradually decreased down to 250–700 regulated genes at 21 dpi, demonstrating that damaged muscles gradually return to normal physiological conditions within the 21 days of repair. In addition to the prominent effect of time on gene expression patterns, samples were separated according to model (*i.e.* Glycerol *vs*. CTX) along the 2^nd^ and 3^rd^ component of the PCA (figure 3A). The vast majority of genes were commonly regulated in both models, but glycerol also induced a specific set of genes while very little genes were specifically regulated by CTX (figure 3B, Table S2). Altogether, these data demonstrate that glycerol and CTX regulate similar pathways during regeneration for which time after injury is the key factor. However, glycerol also triggers a specific set of genes which likely accounts for additional biological processes.

### Glycerol specifically triggers a stronger expression of anti-inflammatory cytokines and activates adipogenic networks while repressing fatty acid oxidation

**Figure 4:**
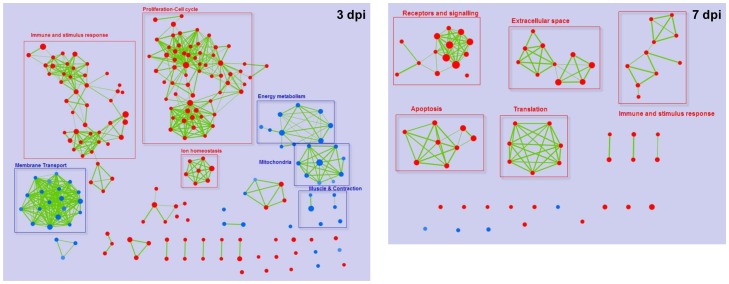
Gene set enrichment mapping of glycerol- *vs.* CTX-injected muscle. Gene set enrichment analysis was performed on glycerol-injected compared to CTX-injected muscles 3 and 7 days after injection, and clustered according to gene set ontology. The size of nodes is proportional to the number of genes contained in the gene set. Red nodes: gene sets upregulated in glycerol *vs*. CTX model, blue nodes: gene sest downregulated in glycerol *vs*. CTX model, green bar: link between two gene sets sharing regulated genes.

In order to assess the main biological functions of the numerous genes regulated in muscle after glycerol and CTX-injections, we performed a Gene Set Enrichment Analysis (GSEA) for the 3 dpi and 7 dpi time points, and analyzed the different sets of genes that were specifically enriched in glycerol- *vs*. CTX-treated muscles. The significantly enriched genesets were analyzed using the enrichment map tool and plugged into Cytoscape, a platform used to visualize complex networks with integration of p-value data. Using this approach, genesets sharing a significant number of genes are connected, generating a summarized network view of the specific biological processes that are differentially regulated between the 2 conditions (figure 4). Consistent with observations at the individual gene level, more gene sets were differentially regulated between glycerol- and CTX-injected muscles at 3 dpi than at 7 dpi, demonstrating that the differential responses of muscles upon glycerol- and CTX-induced injuries initiate early on during the regenerative process.

The strong regulation of sets of genes involved in immune and stimulus response (figure 4) was consistent with the crucial role of inflammation in the muscle regeneration process [4], [6]. Indeed, 3 days after glycerol injection, muscles were characterized by a stronger signature for immune cells and proliferation/cell cycle than after CTX injection, and the differences between both models were partially (immune cell gene sets) or totally (proliferation gene sets) attenuated at 7 dpi (figure 4).

**Figure 5:**
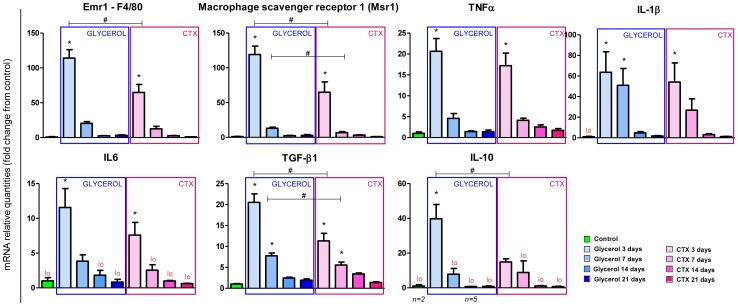
The inflammatory signature is stronger in response to glycerol than to CTX. qPCR analysis of the mRNA levels of various macrophage markers and cytokines. Data are expressed as mean ± s.e.m., n = 5–6/group. * p-value <0.05 *vs*. control, # p-value <0.05 in Glycerol *vs*. CTX at same time points. Emr1; EGF-like module containing mucin-like hormone receptor 1; TNFα, tumor necrosis factor alpha, IL, interleukin; TGF-β1, transforming growth factor beta 1.

The importance of the sequential involvement of pro-inflammatory macrophages (or M1) and anti-inflammatory macrophages (or M2) for muscle repair after an injury is well established [7], [8]. In order to investigate if the inflammatory response is differentially involved in both models after injury, we further analyzed inflammatory molecular signatures in muscle using qPCR. As expected, the mRNA levels of macrophage markers, such as Emr1/F4/80, which is also expressed in eosinophils [37], and the macrophage scavenger receptor MSR-1 massively increased at 3 dpi, and then rapidly decreased at 7 dpi (figure 5A). Interestingly, Emr-1 and MSR-1 were more strongly induced by glycerol than CTX with a 115–120-fold increase in the glycerol model and only 65-fold increase in the CTX model, suggesting that macrophages could trigger a stronger inflammatory response to glycerol injury and thereby potentially exacerbate glycerol-induced adipogenesis. We also analyzed different cytokine expression profiles (figure 5A). As expected, both pro-inflammatory cytokines such as the tumor necrosis factor alpha (TNFα), the interleukins 1 beta (IL-1β) and 6 (IL6) and anti-inflammatory cytokines such as the transforming growth factor beta 1 (TGF-β1) and the interleukin 10 (IL10) were detected in muscle 3 days after injection of glycerol and CTX, potentially resulting from an active presence of M1 and M2 macrophages in injected muscles. However, no IL-4/IL-13 signal was detected in muscles at the mRNA levels (results not shown). The similar expression of pro-inflammatory cytokines in muscles at 3 and 7 dpi in both models demonstrates that both models trigger a similar early inflammatory response. In contrast, glycerol-injected muscles had a stronger anti-inflammatory cytokine mRNA expression as TGF- β1 and IL-10 inductions were approximately 2 fold higher in response to glycerol *vs*. CTX, suggesting that glycerol may elicit stronger M2 macrophage activation. These cytokine mRNA profiles data illustrate that glycerol induces a higher expression of anti-inflammatory cytokines a few days after injury. These results likely illustrate a different extent of inflammatory response between both models, which could influence the cytokine balance and confound signals sent to satellite cells, myoblasts and adipocyte progenitors, and possibly influence adipogenesis.

**Figure 6:**
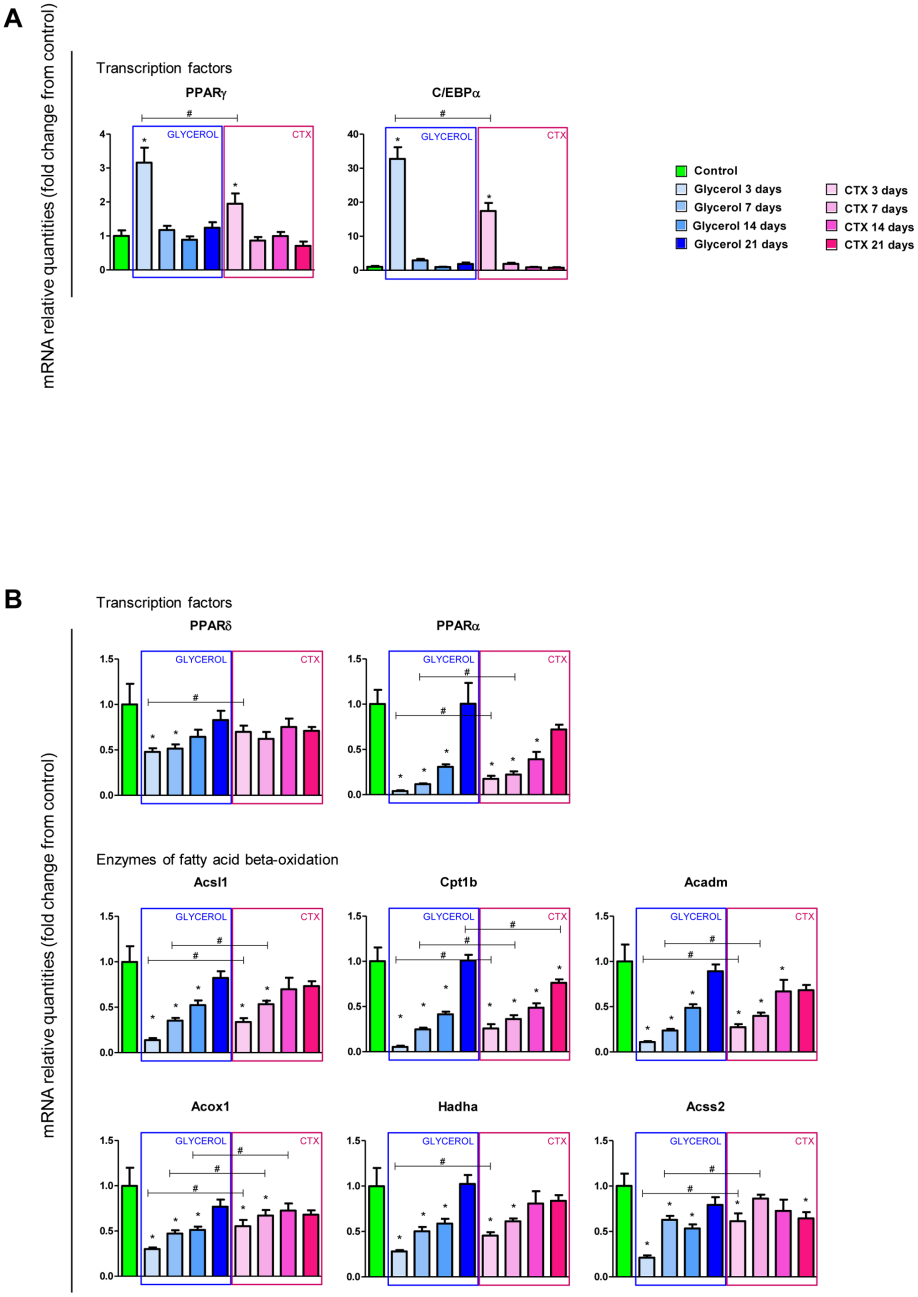
Adipogenesis and β-oxidation are differentially regulated in muscle after glycerol or CTX injection. qPCR analysis of the mRNA levels of different adipogenic (A), or in fatty-acid oxidation (B) regulators. Data are expressed as mean ± s.e.m., n = 5–6/group. * p-value <0.05 *vs*. control, # p-value <0.05 in Glycerol *vs*. CTX at same time points. Acadm, acyl-CoA dehydrogenase medium; Acs/l, acyl-CoA synthesase short-/long-chain; Acss, Acetyl-coenzyme A synthetase; Acox, Acyl-coenzyme A oxidase, Palmitoyl; C/EBP: CCAAT/ Enhancer binding protein; Cpt, carnitine palmitoyltransferase; Hadh, hydroxyacyl-CoA dehydrogenase; PPAR, peroxisome proliferator activated receptor.

Many gene sets involved in muscle contractile function, ion and metabolite transport (membrane transport gene sets), and muscle metabolism were down regulated in glycerol- *vs*. CTX-injected muscles 3 days after injection (figure 4), indicating a stronger molecular signature for muscle and metabolic impairment in the glycerol model. To further characterize the difference between both muscle regeneration models at the molecular level, we performed a pathway analysis of the genes specifically or differentially regulated by glycerol, which highlighted that adipogenesis and fatty acid oxidation are differentially regulated by glycerol and CTX at 3 dpi (figures S2, S3 and S4). The most prominent findings were confirmed by qPCR at the single gene level. Among these, the Peroxisome Proliferator Activated Receptors (PPARs) were preferentially regulated in the glycerol model. PPARγ, the master adipogenic regulator [38], [39], was strongly over-expressed at 3 dpi in glycerol- *vs*. CTX-injected muscles with a 3.2-fold increase in the glycerol model vs 1.6-fold increase only in response to CTX, suggesting a prominent engagement of glycerol-activated adipocyte progenitors in the adipogenic program (figure 6A). Concomitantly, the transcription factor CCAAT/enhancer binding protein α (C/EBPα) was also more strongly activated by glycerol than CTX at 3 dpi (32- *vs*. 17-fold activation, respectively). As adipogenic differentiation is controlled by a transcriptional cascade which relies extensively on PPARγ and C/EBPα [38]–[40], we analyzed downstream functional adipogenic markers.

In contrast, both PPARα and PPARβ/δ, which are prominently expressed in skeletal muscle and control the expression of genes involved in fatty acid catabolism [41], [42], were down-regulated in glycerol- *vs*. CTX-injected muscles (figure 6B, figures S2, S3, S4). In particular, the acyl-CoA synthesase long-chain 1 and short-chain 2, carnitine palmitoyltransferase 1, acyl-CoA, dehydrogenase medium, acyl-coA oxidase 1 and hydroxyacyl-CoA dehydrogenase were dramatically down-regulated 3 days after injection. Their expression gradually increased during muscle regeneration, and muscles almost recovered the basal mRNA level 21 days after injury. Interestingly, glycerol induced a stronger down-regulation of all 6 genes than CTX with a decrease of 70-95%, whereas CTX induced a decrease of only 40–75%, illustrating a differential metabolic shift occurring in response to glycerol or CTX injection.

Altogether, the microarray-based expression data and subsequent qPCR validation confirm that glycerol induces stronger adipogenic differentiation which may relate to the stronger up-regulation of some cytokines and immune cell markers in glycerol injected muscles, and to the increased expression of adipogenic regulators concomitant with a down-regulation of genes controlling fatty acid oxidation. The data also highlight that other biological processes which are differentially regulated in glycerol vs. CTX model at the mRNA level, such as immune response, cell proliferation and muscle metabolism, may also influence ectopic adipogenesis during muscle regeneration.

## Discussion

Intramuscular adipogenesis has emerged as a physiopathological condition of growing interest, due to the correlation between ectopic fat in skeletal muscle and the reduction of muscle function and metabolic homeostasis [43]–[45]. In the present study, we have compared the molecular and cellular processes occuring during glycerol- and CTX-induced regeneration. Both glycerol and CTX induce acute muscle degeneration followed by efficient muscle repair with similar time courses, when assessed at the histological level or by the Principal Component Analysis (PCA) of the global gene expression changes measured in both models. Consistent with the well described mechanisms of muscle regeneration [4], [6], [46], both models induced the transient loss of adult myosin heavy chains (MYH), which recovered after temporary compensation by embryonic MYH. Satellite cell activation also lead to similar activation of the myogenic trasncription factors MyoD1, Myf5, Myogenin. Despite similar or slightly lower levels of degeneration at the histological level in glycerol than CTX-injected muscle, gene set enrichment analysis suggested that the molecular and cellular remodeling during degeneration could be stronger upon glycerol injection. Although the basis to this observation remains unclear, one possibility is that glycerol and CTX may differentially damage the cell membrane and the intracellular components.

Several studies have shown that PDGFRα-positive fibro adipogenic progenitors (FAPs) proliferate upon muscle injury and differentiate into ectopic adipocytes in muscle under certain physio-pathological conditions [20], [21], [47]. As previously reported [16], [17], glycerol-induced regeneration promoted FAP proliferation and ectopic adipocyte formation in muscle in our experiments. Previous studies have shown that adipogenic potential, and more recently FAP proliferation, are induced in various models of muscle injury [17], [48]. It was, however, suggested that the muscle environment is permissive to terminal adipogenic differentiation in muscles injected with glycerol but not with CTX [17]. Consistently, we also observed that PDGFRα expression was transiently elevated 3 days after glycerol and CTX injection when the PDGFRα-positive fibro-adipogenic progenitors (FAPs) proliferate. However, our results demonstrate that CTX injection can still transiently prime muscle towards adipogenic commitment, albeit at a lower extent than glycerol. The transient adipogenic activation observed in both models is supported by an activation of the adipogenic transcription factors PPARγ and C/EBPα, limited to the first few days post injury, and the appearance of perilipin-positive cells in muscle. These adipocytes are larger and more persistent in the glycerol than in the CTX model, as the number of perilipin-expressing cells and the fat area gradually decrease in the CTX model during muscle regeneration. Upon muscle injury, activated FAPs express high levels of IL-6 and co-culture experiments have demonstrated that myogenic differentiation was more efficient in the presence of FAPs [20]. Since we observed an adipogenic response in the two regeneration models tested, it is possible that ectopic adipogenesis may be a hallmark of muscle regeneration that could be required for efficient muscle repair.

At the molecular level, glycerol induced a wider genomic signature than CTX, which most likely accounts for the induction of a stronger adipogenic commitment. Gene set enrichment analysis also revealed the exacerbated regulation of various biological processes by glycerol, which was particularly prominent at early time points. Gene sets involved in proliferation/cell cycle and immune response were strongly enriched 3 days after glycerol injection, illustrating that the cell proliferation required to induce the early steps of tissue repair is stronger after glycerol injection and may participate to stronger ectopic adipogenesis. In particular, glycerol-injected muscles were characterized by a stronger anti-inflammatory signature defined by increased TFG-β1 and IL-10 levels, suggesting that the M1 to M2 macrophage transition occurring during regeneration may differ in the two models and thereby differentially affect adipogenesis. Finally, a differential regulation of metabolic pathways also very likely contributes to the stronger adipogenic response induced by glycerol. In particular, gene sets involved in energy metabolism and mitochondrial function, and many enzymes of fatty acid β-oxidation pathways were down-regulated in glycerol-injected muscles. In contrast, a stronger adipogenic signature was prominent in glycerol over CTX muscle. These results therefore strongly suggest that a shift in the balance between fatty acid storage and utilization contributes to stronger ectopic adipogenesis in glycerol-injected muscle. Interestingly, a molecular pathway analysis demonstrated that PPARs could account for the transcriptional integration of this orchestrated metabolic response. PPARγ, the master regulator of adipogenic differentiation [39], was more strongly regulated by glycerol than CTX. In contrast, PPARα and PPARβ/δ, the PPARs primarily involved fatty acid catabolism [41], [42], were down regulated in glycerol- *vs*. CTX-injured muscles.

Altogether, our data demonstrate that transient adipogenic activation is an integral response of skeletal muscle regeneration which is differentially modulated according to physio-pathological conditions through inflammatory and metabolic cues. In addition, these results also provide a comprehensive transcriptomic resource of the genes commonly or differentially regulated during muscle regeneration and ectopic muscle adipogenesis which will likely turn useful for further characterization of these processes.

## Supporting Information

Figure S1 **Effect of glycerol dosage on muscle degeneration.** Control uninjured tibialis anterior muscle, and tibialis anterior muscles injected with 25 μl 25% (v/v), 25 μl 50% (v/v) or 50 μl 50% (v/v) glycerol, or 25 μl 10 μM CTX were sectioned and stained with laminin and DAPI, 3 days after injection (dpi). The total area occupied by laminin-surrounded fibers was measured by histomorphometry.
(PDF)

Figure S2 **Differential regulation of fatty acid beta oxidation pathway in glycerol** ***vs.*** **control models.** Relative gene expression in glycerol-injected muscles vs. control muscles was mapped on Wikipathways. Colors represent log_2_ of Fold Change (logFC); blue, −2<logFC<0; red, 0<logFC<2, blue and red intensity increases with the amplitude of regulation. Each rectangle represents a probe and is separated into 4 sections, describing the fold change values at 3, 7, 14 and 21 dpi as indicated in the legend. Lpl, lipo-protein lipase; Acs/l, acyl-CoA synthesase short-/long-chain; Acad, acyl-CoA dehydrogenase; Hadh, hydroxyacyl-CoA dehydrogenase; Gyk&Gk2, glycerol kinases; Gpd2, mitochondrial gylcerol 3-phosphate dehydrogenase 2; Tpi1, triosephosphate isomerase 1; Crat, carnitine O-acyltransferase; Cpt, carnitine palmitoyltransferase; Chkb, choline kinase β; Slc25a20, solute carrier family 25 (mitochondrial carnitine/acylcarnitine translocase); member 20.
(PDF)

Figure S3 **Differential regulation of fatty acid beta oxidation pathway in CTX** ***vs.*** **control models.** Relative gene expression in CTX-injected muscles vs. control muscles was mapped on Wikipathways. Colors represent log_2_ of Fold Change (logFC); blue, −2<logFC<0; red, 0<logFC<2, blue and red intensity increases with the amplitude of regulation. Each rectangle represents a probe and is separated into 4 sections, describing the fold change values at 3, 7, 14 and 21 dpi as indicated in the legend. Lpl, lipo-protein lipase; Acs/l, acyl-CoA synthesase short-/long-chain; Acad, acyl-CoA dehydrogenase; Hadh, hydroxyacyl-CoA dehydrogenase; Gyk&Gk2, glycerol kinases; Gpd2, mitochondrial gylcerol 3-phosphate dehydrogenase 2; Tpi1, triosephosphate isomerase 1; Crat, carnitine O-acyltransferase; Cpt, carnitine palmitoyltransferase; Chkb, choline kinase β; Slc25a20, solute carrier family 25 (mitochondrial carnitine/acylcarnitine translocase); member 20.
(PDF)

Figure S4 **Differential regulation of fatty acid beta oxidation pathway in glycerol** ***vs.*** **CTX models.** Relative gene expression in glycerol-injected muscles vs. CTX-injected muscles was mapped on Wikipathways. Colors represent log_2_ of Fold Change (logFC); blue, −2<logFC<0; red, 0<logFC<2, blue and red intensity increases with the amplitude of regulation. Each rectangle represents a probe and is separated into 4 sections, describing the fold change values at 3, 7, 14 and 21 dpi as indicated in the legend. Lpl, lipo-protein lipase; Acs/l, acyl-CoA synthesase short-/long-chain; Acad, acyl-CoA dehydrogenase; Hadh, hydroxyacyl-CoA dehydrogenase; Gyk&Gk2, glycerol kinases; Gpd2, mitochondrial gylcerol 3-phosphate dehydrogenase 2; Tpi1, triosephosphate isomerase 1; Crat, carnitine O-acyltransferase; Cpt, carnitine palmitoyltransferase; Chkb, choline kinase β; Slc25a20, solute carrier family 25 (mitochondrial carnitine/acylcarnitine translocase); member 20.
(PDF)

Table S1 **Reference of Taqman probes used for qPCR.**
(DOC)

Table S2 **List of regulated genes in the different models.** Lists of genes are separated in the different tabs by positive or negative regulation and by time points in the different models (Glycerol *vs.* sham, CTX *vs.* Sham or Glycerol *vs.* CTX). Genes with a fold change higher than 2 and an adjusted P-value below 0.01 (Benjamini and Hochberg multiple testing correction) were considered regulated.
(XLS)
