# Supplementary figures and images for: Genomic Profiling Reveals That Transient Adipogenic Activation Is a Hallmark of Mouse Models of Skeletal Muscle Regeneration
Source: PLoS One. 2013 Aug 15;8(8):e71084. doi: 10.1371/journal.pone.0071084 (PMC3744575; doi:10.1371/journal.pone.0071084)

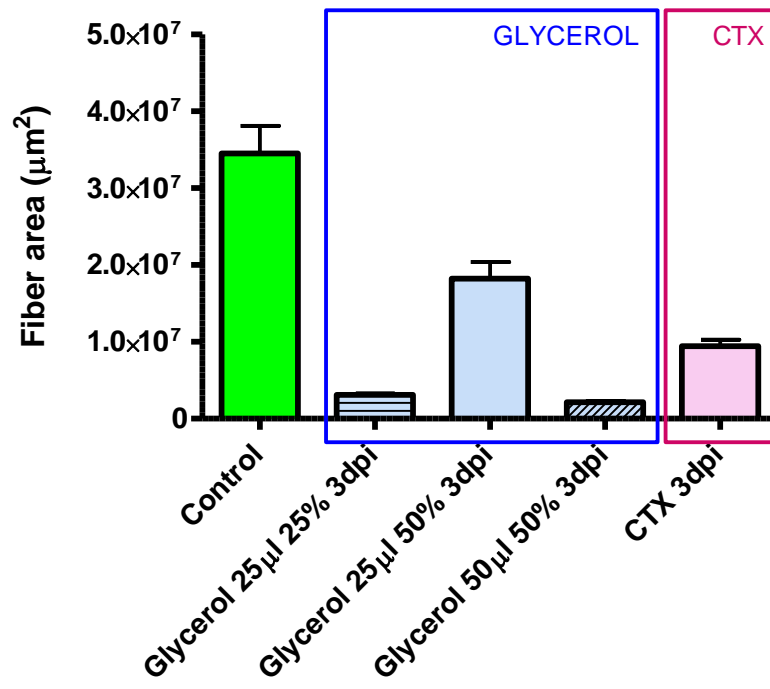

Supplement: Figure S1 — Effect of glycerol dosage on muscle degeneration. Control uninjured tibialis anterior muscle, and tibialis anterior muscles injected with 25 μl 25% (v/v), 25 μl 50% (v/v) or 50 μl 50% (v/v) glycerol, or 25 μl 10 μM CTX were sectioned and stained with laminin and DAPI, 3 days after injection (dpi). The total area occupied by laminin-surrounded fibers was measured by histomorphometry. (PDF) [file pone.0071084.s001.pdf]

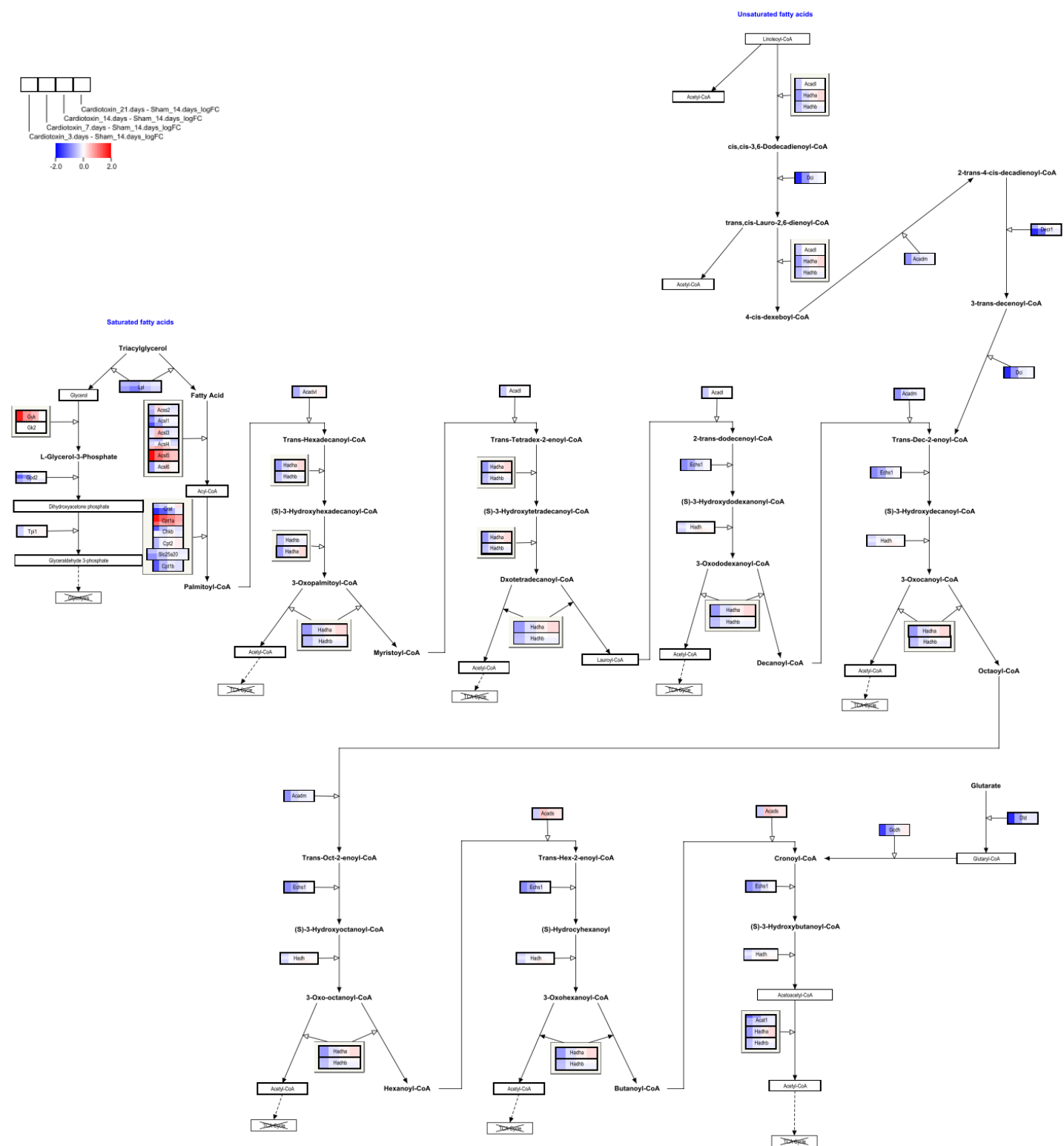

Supplement: Figure S3 — Differential regulation of fatty acid beta oxidation pathway in CTX vs. control models. Relative gene expression in CTX-injected muscles vs. control muscles was mapped on Wikipathways. Colors represent log2 of Fold Change (logFC); blue, −2<logFC<0; red, 0<logFC<2, blue and red intensity increases with the amplitude of regulation. Each rectangle represents a probe and is separated into 4 sections, describing the fold change values at 3, 7, 14 and 21 dpi as indicated in the legend. Lpl, lipo-protein lipase; Acs/l, acyl-CoA synthesase short-/long-chain; Acad, acyl-CoA dehydrogenase; Hadh, hydroxyacyl-CoA dehydrogenase; Gyk&Gk2, glycerol kinases; Gpd2, mitochondrial gylcerol 3-phosphate dehydrogenase 2; Tpi1, triosephosphate isomerase 1; Crat, carnitine O-acyltransferase; Cpt, carnitine palmitoyltransferase; Chkb, choline kinase β; Slc25a20, solute carrier family 25 (mitochondrial carnitine/acylcarnitine translocase); member 20. (PDF) [file pone.0071084.s003.pdf]
