# Supplementary material for: Genomic Profiling Reveals That Transient Adipogenic Activation Is a Hallmark of Mouse Models of Skeletal Muscle Regeneration
Source: PLoS One. 2013 Aug 15;8(8):e71084. doi: 10.1371/journal.pone.0071084 (PMC3744575; doi:10.1371/journal.pone.0071084)

Figure S3. Fatty acid oxidation pathway in glycerol *vs.* CTX.

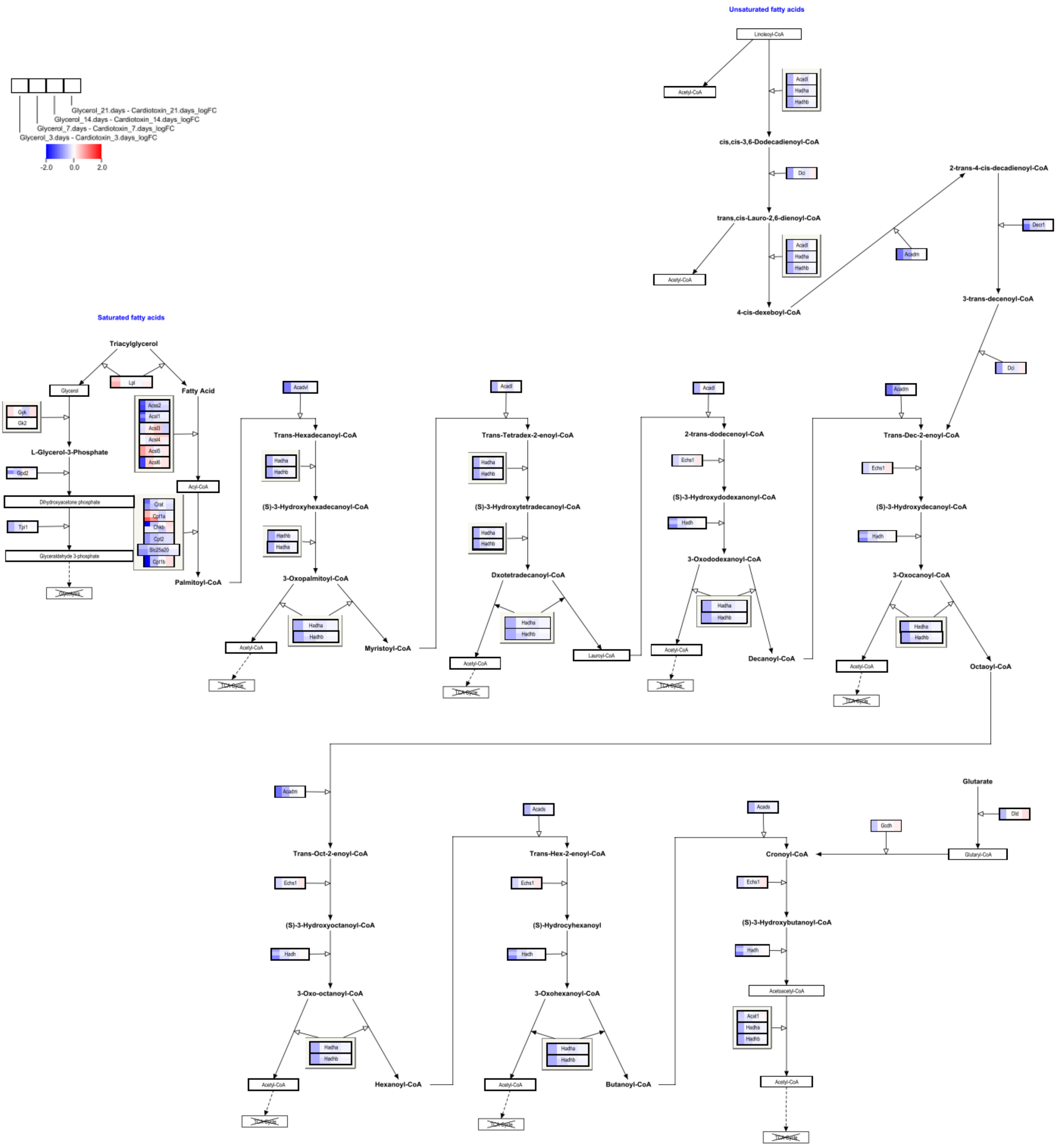

Supplement: Figure S4 — Differential regulation of fatty acid beta oxidation pathway in glycerol vs. CTX models. Relative gene expression in glycerol-injected muscles vs. CTX-injected muscles was mapped on Wikipathways. Colors represent log2 of Fold Change (logFC); blue, −2<logFC<0; red, 0<logFC<2, blue and red intensity increases with the amplitude of regulation. Each rectangle represents a probe and is separated into 4 sections, describing the fold change values at 3, 7, 14 and 21 dpi as indicated in the legend. Lpl, lipo-protein lipase; Acs/l, acyl-CoA synthesase short-/long-chain; Acad, acyl-CoA dehydrogenase; Hadh, hydroxyacyl-CoA dehydrogenase; Gyk&Gk2, glycerol kinases; Gpd2, mitochondrial gylcerol 3-phosphate dehydrogenase 2; Tpi1, triosephosphate isomerase 1; Crat, carnitine O-acyltransferase; Cpt, carnitine palmitoyltransferase; Chkb, choline kinase β; Slc25a20, solute carrier family 25 (mitochondrial carnitine/acylcarnitine translocase); member 20. (PDF) [file pone.0071084.s004.pdf]
