# Supplementary material for: Genomic Profiling Reveals That Transient Adipogenic Activation Is a Hallmark of Mouse Models of Skeletal Muscle Regeneration
Source: PLoS One. 2013 Aug 15;8(8):e71084. doi: 10.1371/journal.pone.0071084 (PMC3744575; doi:10.1371/journal.pone.0071084)
Supplement: Table S1 — Reference of Taqman probes used for qPCR. (DOC) [file pone.0071084.s005.doc]

**Table S1.**

| **Gene** | **Reference** |
| --- | --- |
| 18S rRNA | 4319413E |
| Acadm | Mm01323360_g1 |
| Adipoq | Mm00456425_m1 |
| Acox1 | Mm01246831_m1 |
| Acsl1 | Mm00484217_m1 |
| Acss2 | Mm00480101_m1 |
| Cpt1b | Mm00487200_m1 |
| C/EBPα | Mm01265914_s1 |
| Ear5 | Mm00658916_s1 |
| Emr1-F4/80 | Mm00802529_m1 |
| Exp | Mm00514768_m1 |
| Hadha | Mm00805228_m1 |
| Hadhb | Mm01210656_m1 |
| IL-1β | Mm01336189_m1 |
| IL-4 | Mm00445259_m1 |
| IL-6 | Mm04446190_m1 |
| IL-10 | Mm00439614_m1 |
| IL-13 | Mm00434204_m1 |
| Msr1 | Mm00446214_m1 |
| Myf5 | Mm00435125_m1 |
| Myh4 | Mm01332541_m1 |
| Myh8 | Mm01329494_m1 |
| MyoD1 | Mm00440387_m1 |
| Myogenin | Mm00446194_m1 |
| Pax7 | Mm00834082_m1 |
| PDGF-Rα | Mm00440701_m1 |
| PPARα | Mm00440939_m1 |
| PPARδ | Mm00803184_m1 |
| PPARγ | Mm00440945_m1 |
| Retn | Mm00445641_m1 |
| TGF-β1 | Mm01178820_m1 |
| TNFα | Mm00443258_m1 |
